# Supplementary material for: A carboxylate switch point controls long-range energy transduction in respiratory Complex I
Source: Nat Commun. 2026 Jul 1;17:5737. doi: 10.1038/s41467-026-74767-6 (PMC13324432; doi:10.1038/s41467-026-74767-6)
Supplement: Supplementary file 1 — Supplementary information [file 41467_2026_74767_MOESM1_ESM.pdf]

Supplementary Information

A Carboxylate Switch Point Controls Long-Range Energy Transduction in Respiratory Complex I

Adel Beghiah[1,a], Patricia Saura[1,a], Terezia Kovalova[1,a], Franziska Hoeser[2], Thorsten Friedrich[2], Ville R. I. Kaila[1,*]

[1]Department of Biochemistry and Biophysics, Stockholm University, 10691, Stockholm, Sweden.
[2]Institut für Biochemie, Albert-Ludwigs-Universität Freiburg, Germany
[a] Contributed equally to this work.
[*]Corresponding author: Ville R. I. Kaila, E-mail: ville.kaila@dbb.su.se

Content

Supplementary Fig. 1. Molecular simulation setups.
Supplementary Fig. 2. Disruption of the water wire along the E-channel in the resting state.
Supplementary Fig. 3. Hydration dynamics along the E-channel in Complex I and the D79N^{A} variant.
Supplementary Fig. 4. Electric field effects along the E-channel.
Supplementary Fig. 5. Convergence of the QM/MM free energy simulations.
Supplementary Fig. 6. Protein purification.
Supplementary Fig. 7. Oxygen consumption in cytoplasmic membranes.
Supplementary Fig. 8. Cryo-EM data analysis and validation of WT Complex I.
Supplementary Fig. 9. Cryo-EM data analysis and validation of the D79N^{A} variant.
Supplementary Fig. 10. Example cryo-EM densities of key regions.
Supplementary Fig. 11. Structure of conserved loops.
Supplementary Fig. 12. Water network along the E-channel.
Supplementary Fig. 13. Extended kinetic models.
Supplementary Fig. 14. Multiple sequence alignment of NuoA, NuoH and NuoK.

Supplementary Table 1 | Activity of WT Complex I and the D79N^{A} variant.
Supplementary Table 2 | List of designed primers.
Supplementary Table 3 | List of employed buffers.
Supplementary Table 4 | Cryo-EM data collection, refinement, and validation statistics.
Supplementary Table 5 | List of MD simulations.
Supplementary Table 6 | List of QM/MM simulations.
Supplementary Table 7 | Rate constants used in the kinetic models.

Supplementary references

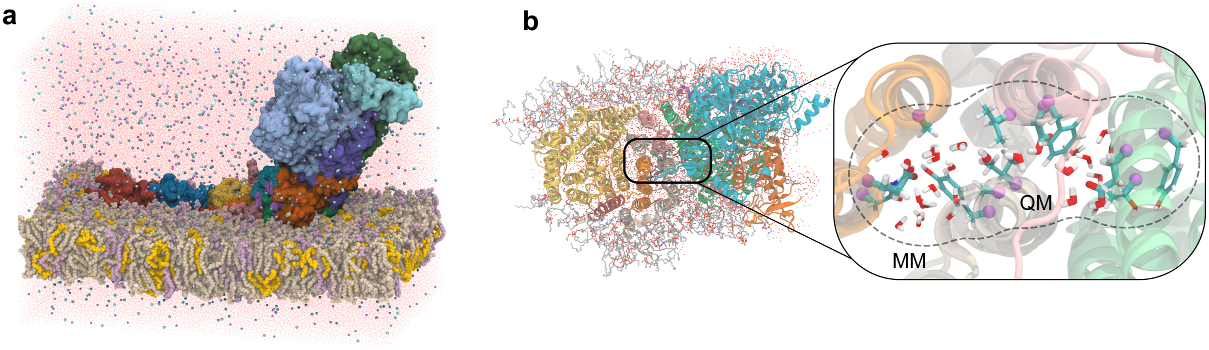

Supplementary Fig. 1 | Molecular simulation setups. a, Setup of the classical MD simulations. **b**, Setup of the QM/MM calculations (*left*), and a closeup of the *ca.* 200 atoms in the QM region (*inset*, *right*). Link atoms are shown as purple spheres.

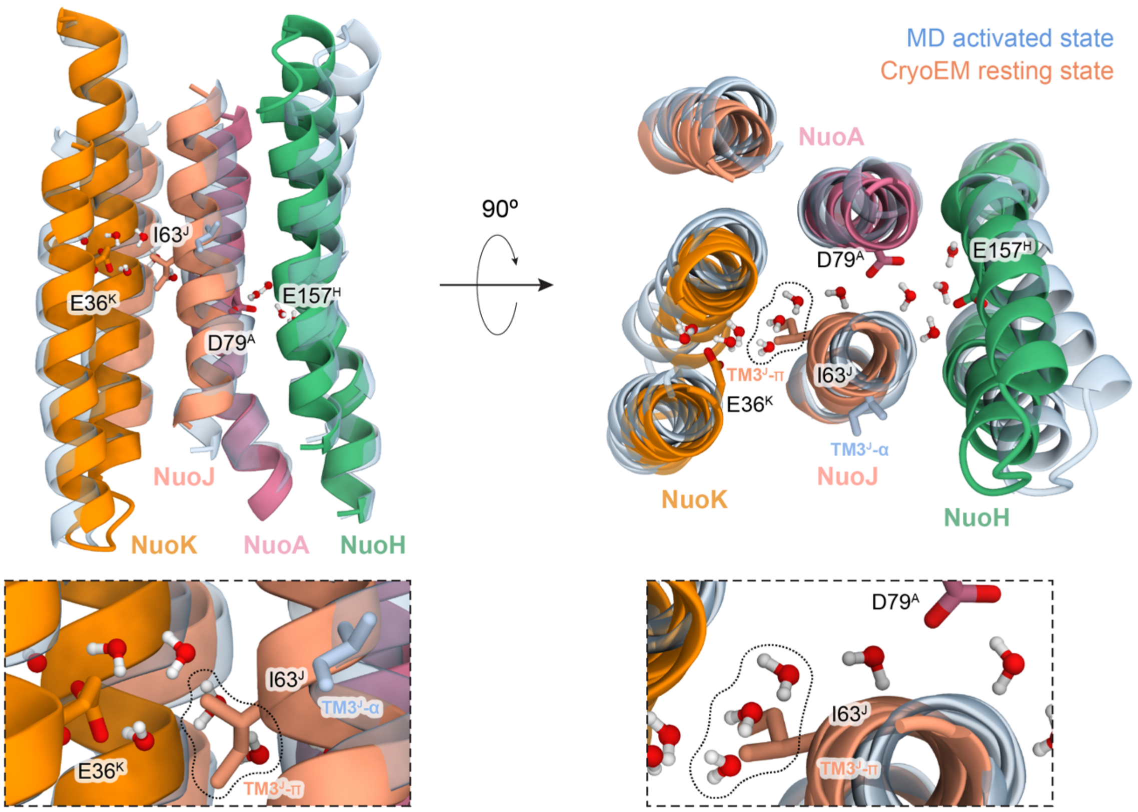

Supplementary Fig. 2. Disruption of the water wire along the E-channel in the resting state.

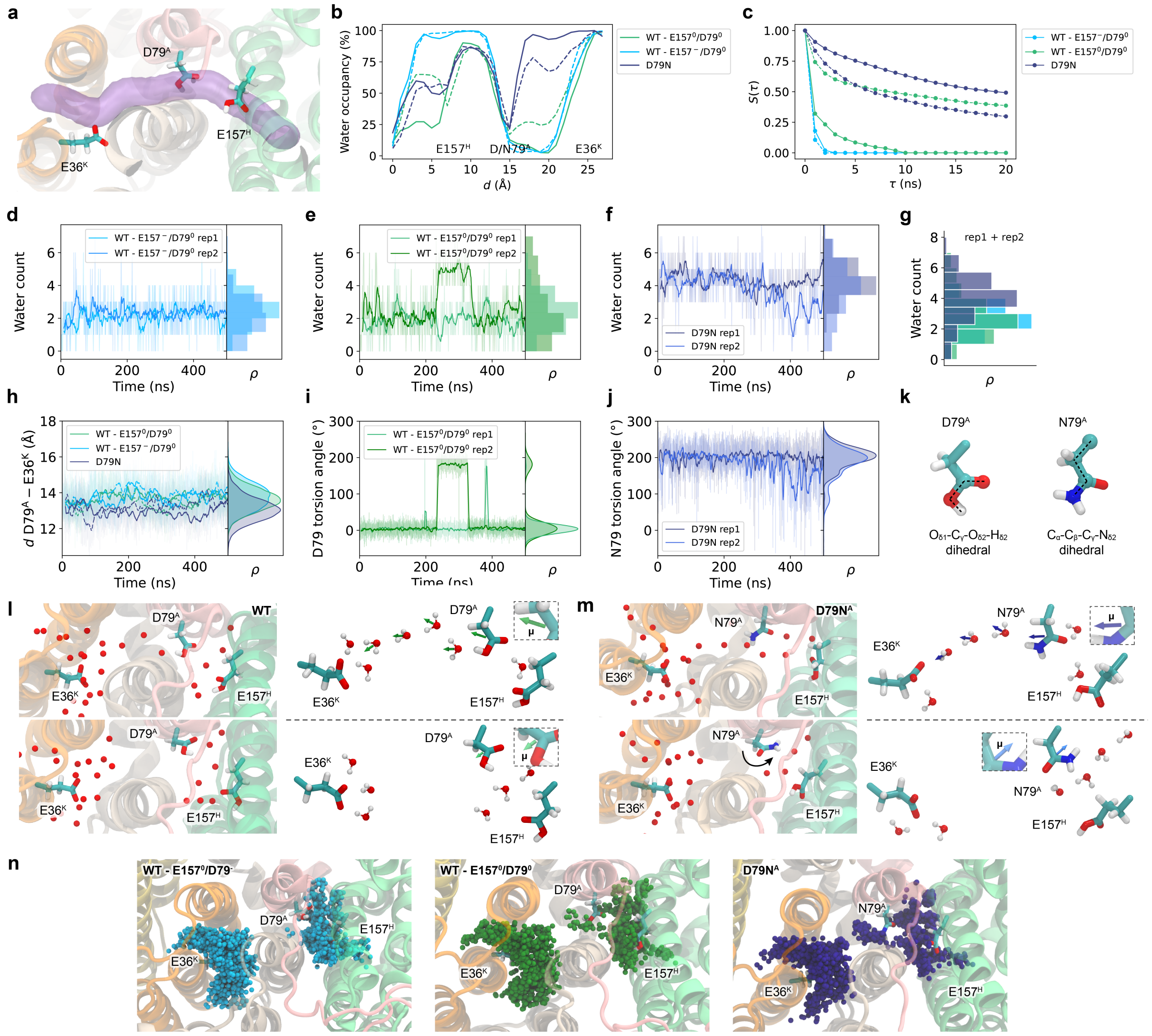

Supplementary Fig. 3 | Hydration dynamics along the E-channel in Complex I and the D79N^{A} variant. a, Proton pathway along E157^{H} via D79^{A} to E36^{K}. **b,** Hydration level along the proton pathway. **c**, Survival probability of water molecules in the region between D79^{A} and E36^{K}. **d-f**, Number of water molecules between E157^{H}/D79^{A} and E36^{K} during MD simulations (**d**, WT with E157^{H} deprotonated; **e**, WT with E157^{H} protonated; **f**, the D79N^{A} variant). **g**, Histograms of the water count from **d-f**. **h**, Dynamics of the D(N)79^{A}–E36^{K} distance during MD simulations. **i**, Dihedral angle of the protonated D79^{A}. The '*trans'* conformation correlates with higher hydration around the region (see panel **e**). **j**, Dihedral angle of N79^{A} in MD simulations of the D79N^{A} variant. The orientation of N79^{A}, with the amine moiety pointing towards E36^{K}, correlates with a higher hydration level (see panel **f**) relative to the other orientation, with the carbonyl group pointing towards E36^{K}. **k,** Definition of the dihedral angles shown in **i** and **j**. **l,** *Left:* Snapshots from MD simulations of the WT, showing different hydration patterns with different D79^{A} side-chain configurations, and *right*: dipole moment (μ, green arrows) of D79^{A} (and key water molecules) in the different configurations. Dipole moments are represented in elementary charge units · Å. **m**, *Left:* Snapshots from MD simulations of D79N^{A}, showing the two sidechain orientations of N79^{A}, and the alternated hydration level. Both states exchange on the simulation timescale. *Right*: Dipole moments of N79^{A} and key water molecules (μ, blue arrows) with different orientations of N79^{A}. **n**, Top view of MD-averaged hydration of the proton pathway between E157^{H}/D79^{A} and E36^{K}. *Left*: WT Complex I with E157^{H} deprotonated, *middle*: WT Complex I with E157^{H} protonated, *right:* the D79N^{A} variant. Data are provided in the Source Data file.

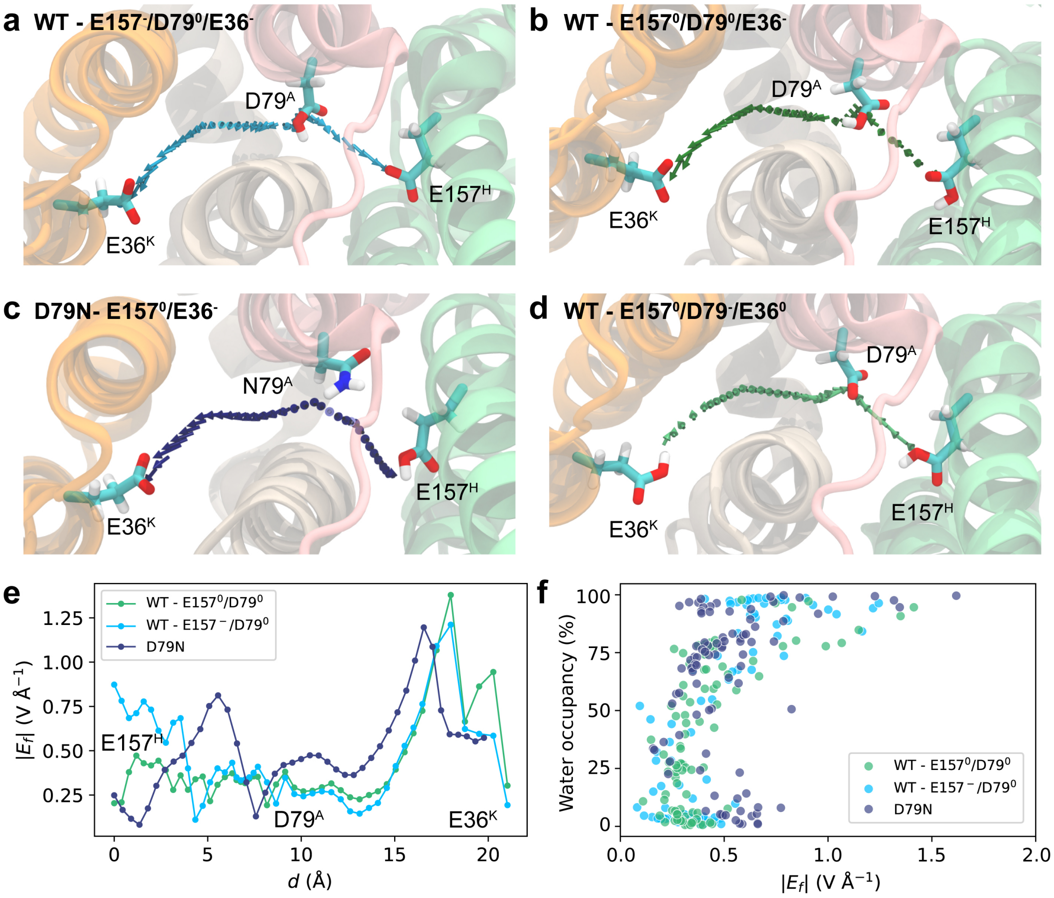

Supplementary Fig. 4 | Electric field effects along the E-channel. a, WT with E157^{H} deprotonated; **b**, WT with E157^{H} protonated; **c**, D79N^{A} variant, and **d**, WT after proton transfer from D79^{A} to E36^{K}, with E157^{H} protonated. The average electric field (E_f) was computed from the classical MD simulations (average from 1 frame/ns). **e**, Electric field strength along the proton pathway. **f**, Correlation of the electric field strength and the water occupancy along the pathway. Data are provided in the Source Data file.

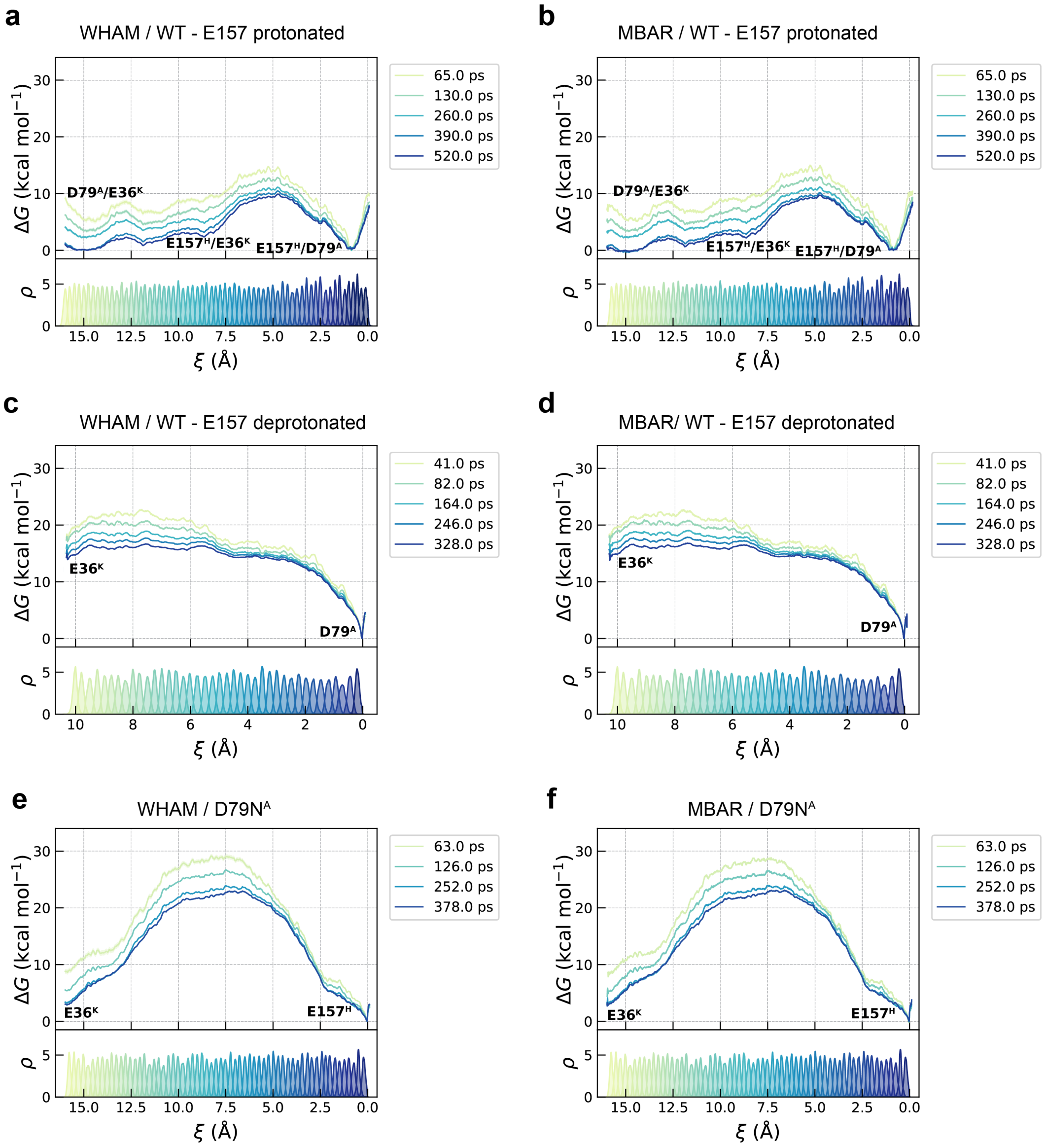

Supplementary Fig. 5 | Convergence of the QM/MM free energy simulations. Convergence of the free energy profiles at different levels of sampling (*top*) and histogram distributions of the QM/MM-US windows along the reaction coordinates (*bottom*) in **a**, WT with $E157^{H}$ protonated; **b**, WT with $E157^{H}$ deprotonated, and **c**, the $D79N^{A}$ variant. Data are provided in the Source Data file.

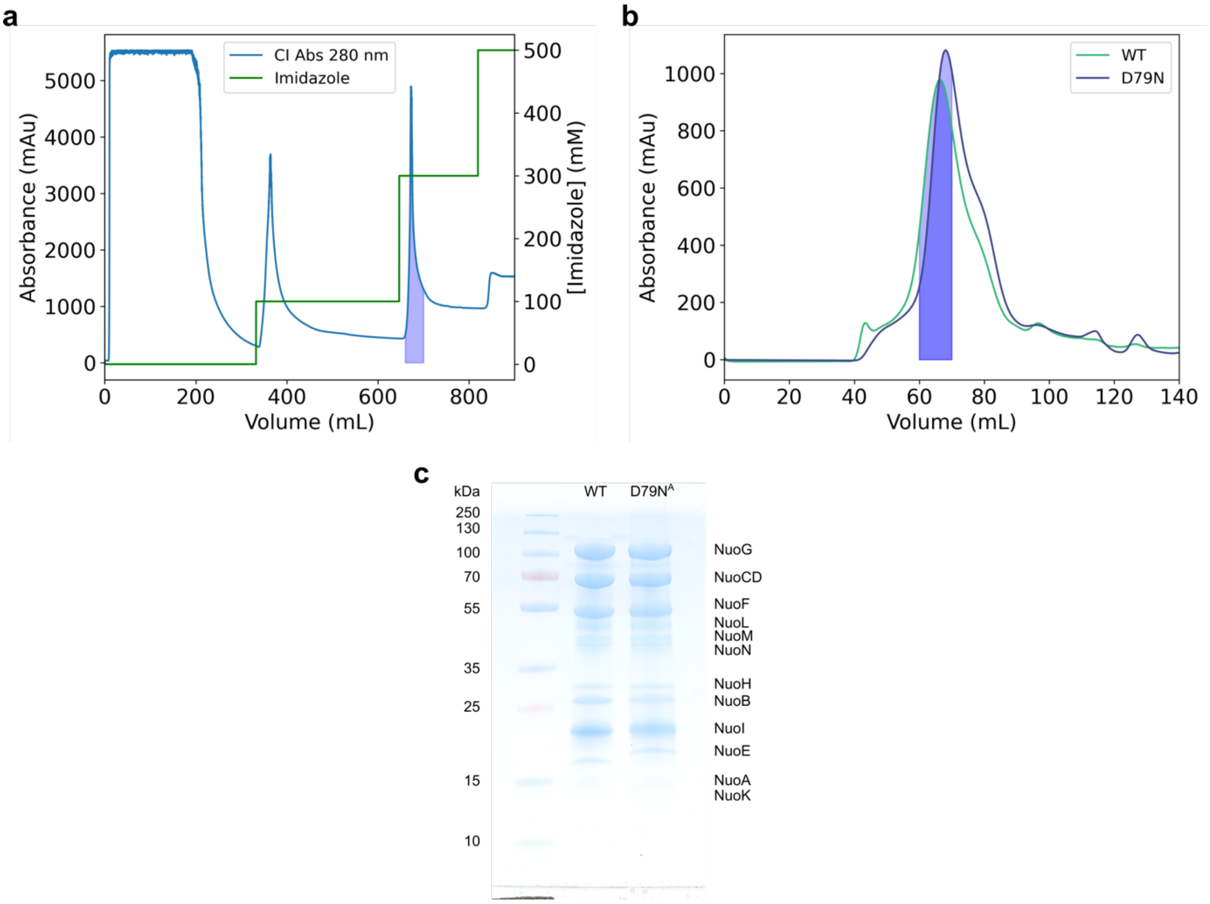

Supplementary Fig. 6 | Protein purification. **a**, Affinity chromatography profile monitored at 280 nm (*blue*) and imidazole concentration (*green*). Fractions collected upon elution with 300 mM imidazole are indicated by the integrated blue surface under the absorbance signal. **b**, Size exclusion chromatography profile monitored at 280 nm for Complex I (*green*) and the D79N^{A} variant (*purple*). Collected fractions are shown by the integrated surface under the absorbance signal. **c**, SDS-PAGE fraction of purified Complex I and the D79N^{A} variant. Data are provided in the Source Data file.

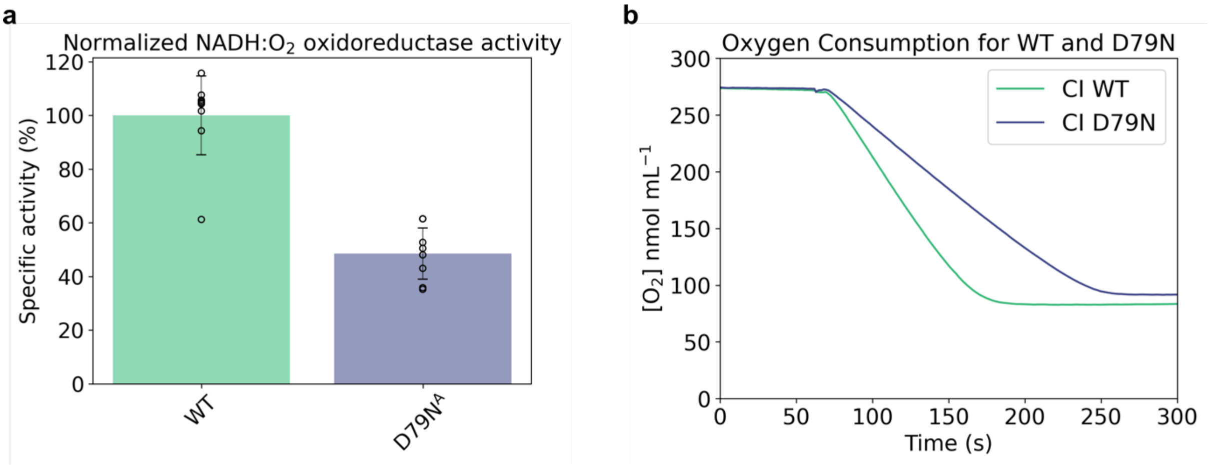

Supplementary Fig. 7 | Oxygen consumption in cytoplasmic membranes. a, Normalised oxygen consumption activity in cytoplasmic membranes from *E. coli* expressing the Complex I (100% activity corresponding to 0.55 ± 0.12 µmol min^{-1} mg^{-1}) and the $D79N^A$ variant. **b**, Raw data of the oxygen consumption of membranes expressing Complex I and the $D79N^A$ variant (250-300 µg of protein). Data are provided in the Source Data file.

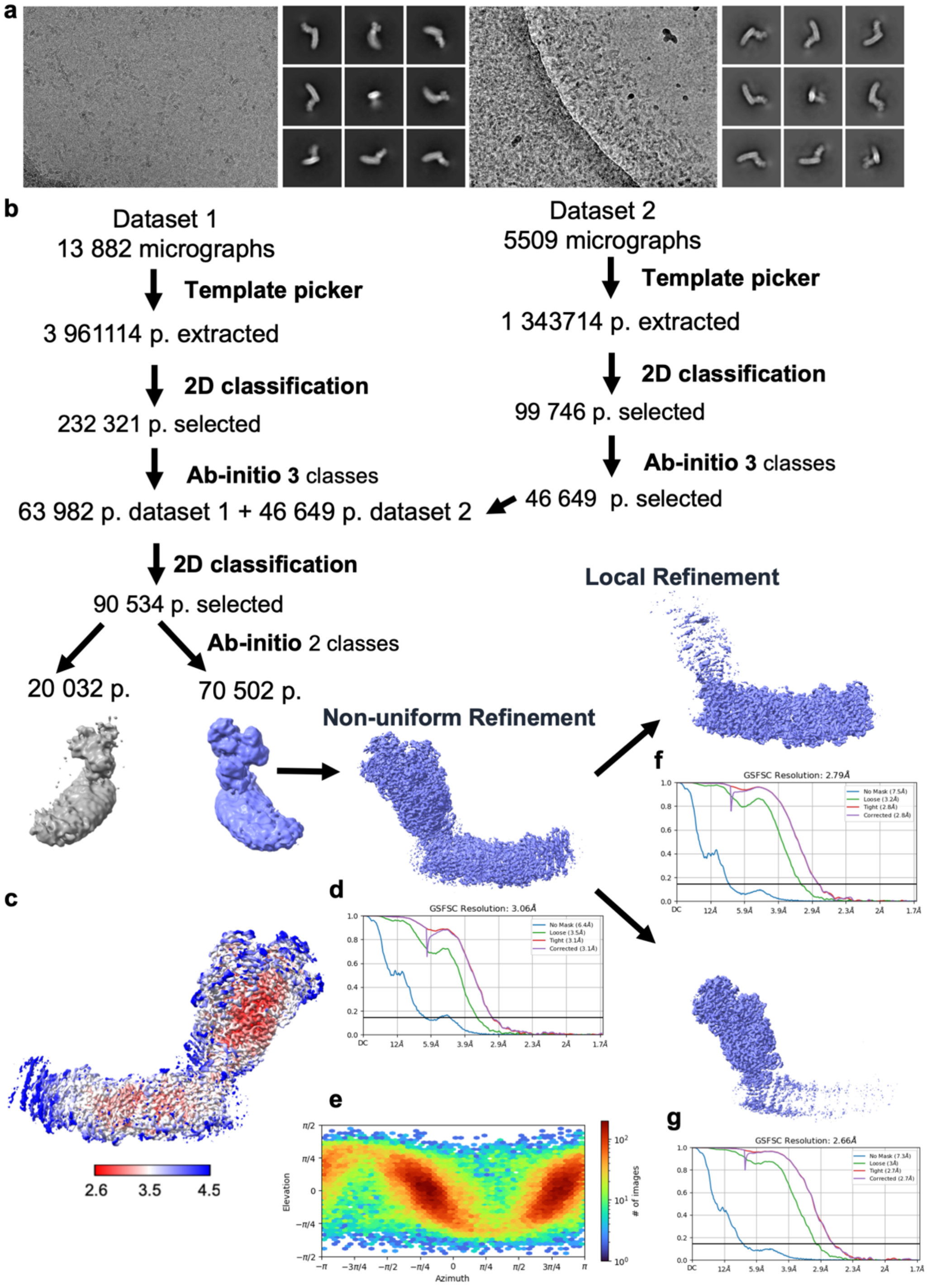

Supplementary Fig. 8 | Cryo-EM data analysis and validation of Complex I. **a**, Example of micrographs showing particle distribution for dataset 1 and 2 complemented with example of the 2D classification with various views. **b**, Schematic overview of data processing work flow. **c**, Local resolution representation of the non-uniform refined map of WT Complex I. **d**, Fourier shell correlation (FSC) curve of the non-uniform refined map of WT Complex I corrected for the effects of masking. **e**, Distribution of particle orientations of the non-uniform refinement of WT Complex I. **f**, Fourier shell correlation (FSC) curve of the local refinement of the membrane domain of WT Complex I corrected for the effects of masking. **g**, Fourier shell correlation (FSC) curve of the local refinement of the hydrophilic domain of WT Complex I, corrected for the effects of masking.

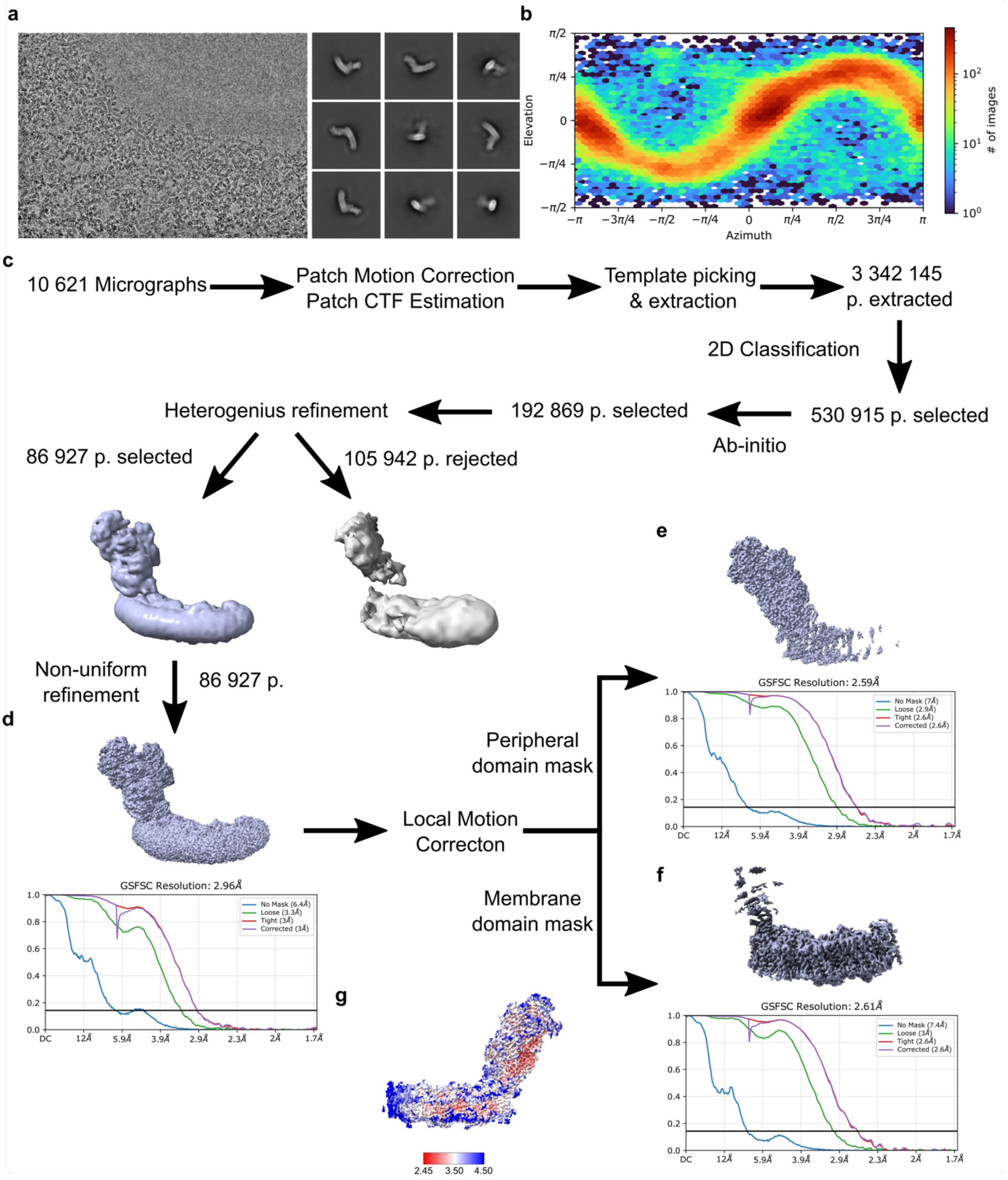

Supplementary Fig. 9 | Cryo-EM data analysis and validation of the D79N^{A} variant. a, Example of micrograph showing particle distribution and 2D classification in various views. **b**, Distribution of particle orientations of the non-uniform refinement of the D79N^{A} variant. **c**, Processing overview performed on CryoSparc v4.4.0. **d**, Fourier shell correlation (FSC) curve of the non-uniform refined map corrected for the effects of masking. **e**, Fourier shell correlation (FSC) curve of the local refinement of the membrane domain corrected for the effects of masking. **f**, Fourier shell correlation (FSC) curve of the local refinement of the hydrophilic domain, corrected for the effects of masking. **g**, Local resolution estimation representation of the non-uniform refined map.

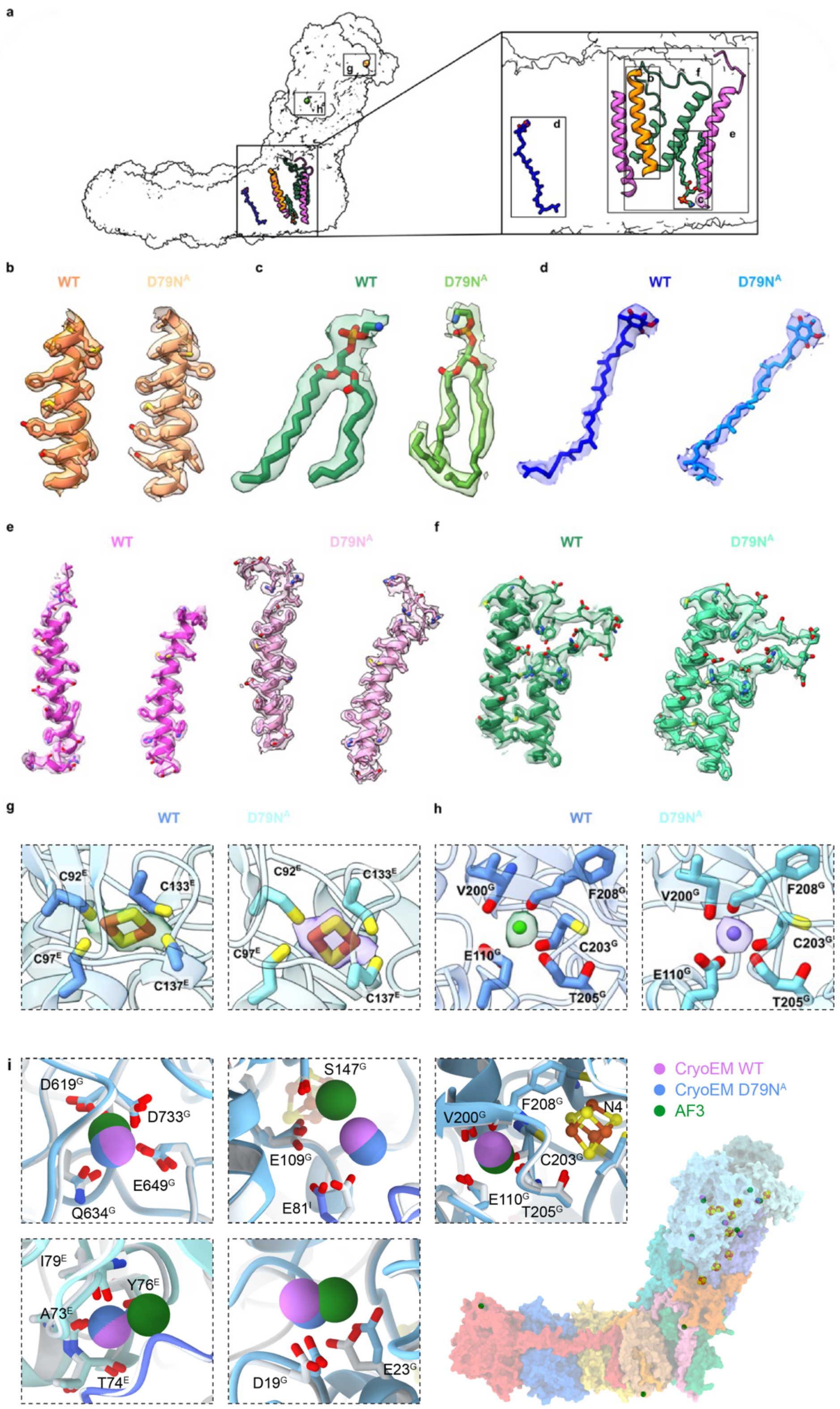

Supplementary Fig. 10 | Example cryo-EM densities of key regions. **a**, Location of regions shown in a-h. **b**, TM3^J. **c**, PE lipid (3PE) near NuoH. **d**, Membrane-bound ubiquinone-8 near NuoN. **e**, TM1-2 of NuoA, with an unresolved loop between residues S45^A and D55^A. **f**, TM5-6 loop of NuoH. **g**, FeS centre (N1a) located in the NuoE subunit. **h**, Ca^{2+} ion located in the NuoG subunit. **i**, Resolved Ca^{2+} ions from cryo-EM structure (WT in *purple*, D79N^A in *blue*), and based on AF3 prediction (in *green*).

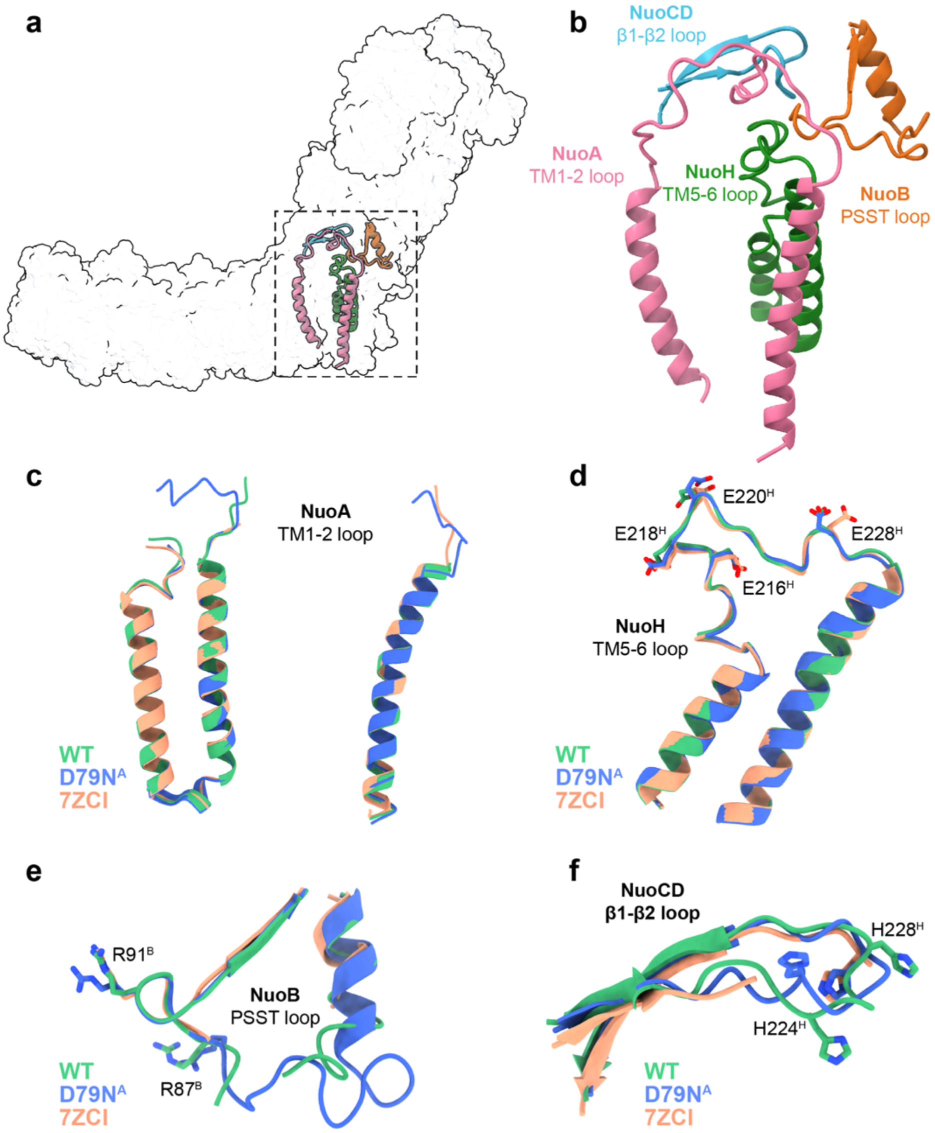

Supplementary Fig. 11 | Structure of conserved loops. a, Location of visualised regions. **b**, Closeup of the loops. Structure of **c**, TM1-2 of NuoA, **d**, TM5-6 loop of NuoH, **e**, PSST loop of NuoB, **f**, β1-β2 loop of NuoCD for Complex I and the D79N^{A} variant, compared to a previous resting state structure of Complex I (PDB ID:7ZCI).

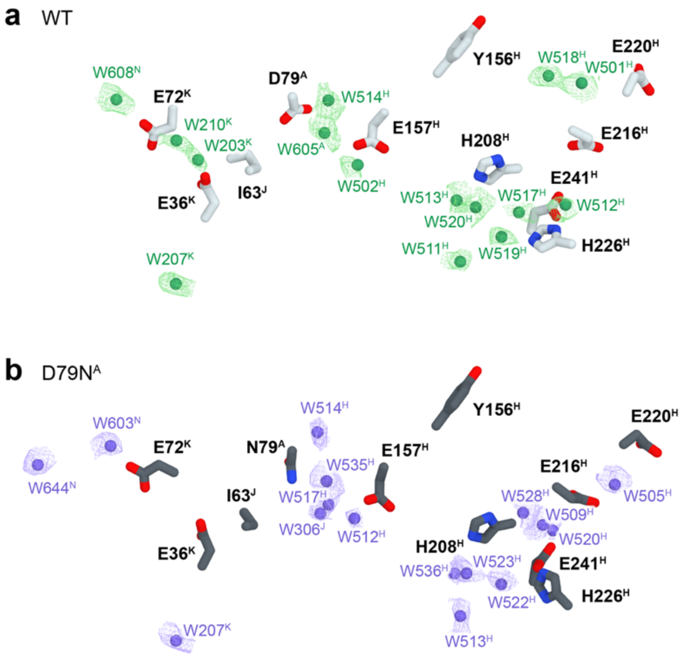

Supplementary Fig. 12. Water network along the E-channel in a, WT Complex I (PDB ID: 9TAK), and b, the D79N^{A} variant (PDB ID: 9TAO).

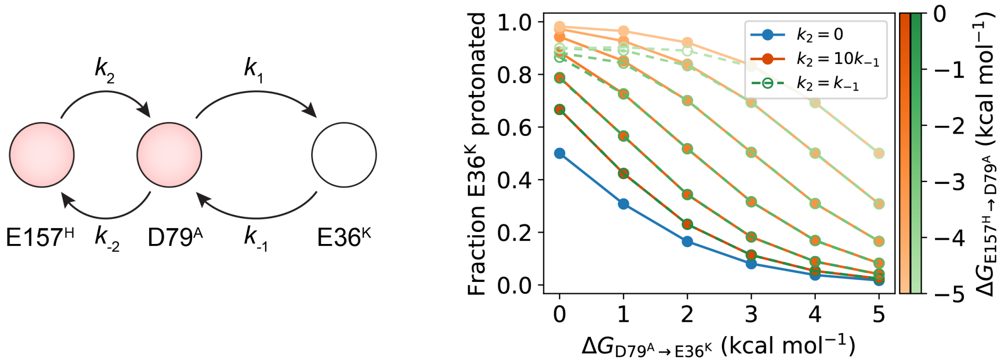

Supplementary Fig. 13. Extended kinetic models, probing how the rate of back-transfer (see Supplementary Table 7 for the rate constants) affects the protonation fraction of $E36^K$ under different driving forces. Data are provided in the Source Data file.

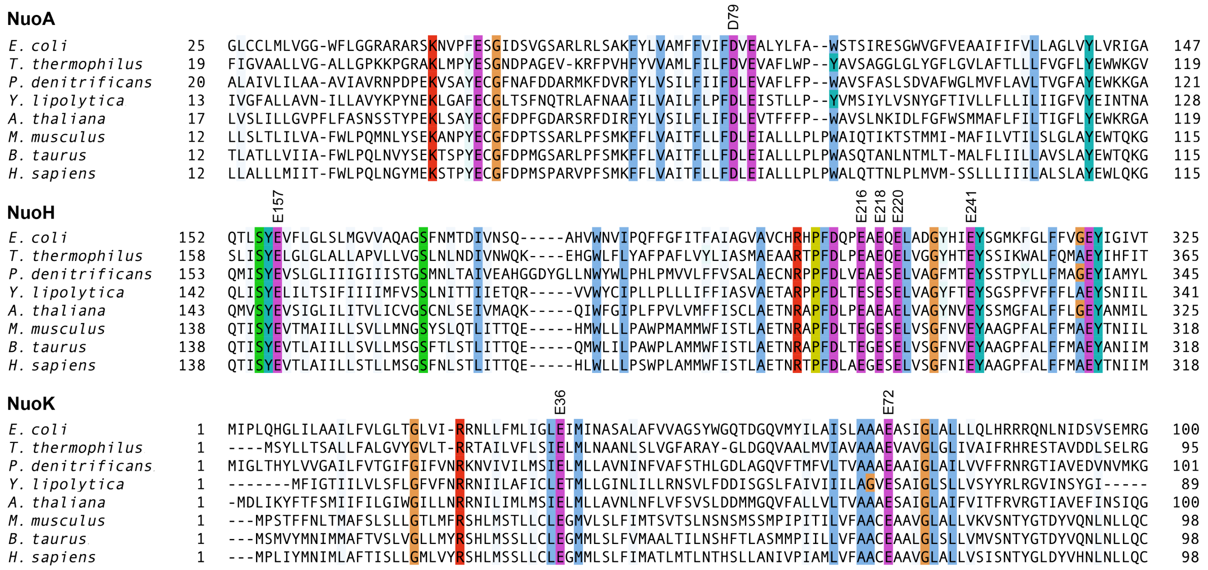

Supplementary Fig. 14. Multiple sequence alignment (MSA) of NuoA, NuoH and NuoK, highlighting residues around the E-channel region. Coloured residues represent up to 90% of conservation, using the ClustalX colouring scheme. The MSA was performed with ClustalΩ[1].

Supplementary Table 1 | Activity of Complex I (WT) and the D79N^A variant.

	Proton pumping activity				
	**ΔpH**		**Δψ**		**Orientation**
	Rfu	% of WT	$\Delta Abs_{588-625}$	% of WT	%
**WT**	0.81 ± 0.06	100% ± 7.0%	0.025 ± 0.003	100% ± 10.9%	83.9% ± 7.4%
**D79N^A**	0.15 ± 0.02	18.5% ± 2.7%	0.0056 ± 0.0009	22.3% ± 3.3%	88.9% ± 2.6%
	**Oxidoreductase activity**				
	**NADH:O_2 activity (in membranes)**			**NADH:DQ activity (in detergent)**	
	μmol min^{-1} mg^{-1}	% of WT	Normalised to FeCN	μmol min^{-1} mg^{-1}	% of WT
**WT**	0.55 ± 0.12	100% ± 22.6%	100% ± 14.7%	28.8 ± 2.41	100% ± 8.35%
**D79N^A**	0.33 ± 0.08	59.9% ± 14.8	48.5% ± 9.5%	7.57 ± 1.54	26.2% ± 5.35%

Supplementary Table 2 | List of designed primers. Modified codons in bold, exchanged bases in italics. Restriction sites with silent mutations (in *italics*) are underlined.

Oligonucleotide	Sequence
nuoA_D79N_fwd	5'-CCATGTTCTTCGTTATCTTC***AAC***GTTGAAGC*TT*TGTATCTGTTCGCATGGTC-3'
nuoA_D79N_rev	5'-GACCATGCGAACAGATACAAAGCTTCAACGTTGAAGATAACGAAGAACATGG-3'
seq_nuoA_D79N	5'-GAGGTCGAAAAACGTG-3'

Supplementary Table 3 | List of employed buffers.

**Cell resuspension buffer**	50 mM MES pH 6.0, 50 mM KCl
**Membrane resuspension buffer**	50 mM MES pH 6.0, 50 mM KCl, 5 mM $MgCl_2$, 10% glycerol
**IMAC buffer A**	50 mM MES pH 6.0, 50 mM KCl, 5 mM $MgCl_2$, 10% glycerol, 0.005% LMNG, 20 mM Imidazole
**IMAC buffer B**	50 mM MES pH 6.0, 50 mM KCl, 5 mM $MgCl_2$, 10% glycerol, 0.005% LMNG, 500 mM Imidazole
**SEC buffer C**	50 mM MES pH 6.0, 50 mM KCl, 5 mM $MgCl_2$, 10% glycerol, 0.005% LMNG
**Cryo-EM buffer**	50 mM MES pH 6.0, 150 mM KCl, 5 mM $MgCl_2$, 2% glycerol, 0.005% LMNG
**Reconstitution buffer**	50 mM MES pH 6.7, 150 mM NaCl
**FeCN buffer**	50 mM MES pH 6.7, 150 mM NaCl, 1 mM $K_3Fe(CN)_6$
**ACMA buffer**	50 mM MES pH 6.7, 150 mM NaCl, 4 µM ACMA
**Oxonol VI buffer**	50 mM MES pH 6.7, 150 mM NaCl, 5 µM oxonol, 100 nM monensin, 300 mM mannitol

Supplementary Table 4 | CryoEM data collection, refinement, and validation statistics.

**Data collection, processing**	**Complex I overall** PDB ID: 9TAJ	**Complex I membrane** PDB ID: 9TAK	**Complex I hydrophilic** PDB ID: 9TAL	**D79N^{A} variant overall** PDB ID: 9TAM	**D79N^{A} variant membrane** PDB ID: 9TAO	**D79N^{A} variant hydrophilic** PDB ID: 9TAN
Voltage (kV)	300	300	300	300	300	300
Magnification	105,000	105,000	105,000	105,000	105,000	105,000
Electron exposure (e^{-}/Å^2)	40	40	40	40	40	40
Pixel size (Å)	0.825	0.825	0.825	0.825	0.825	0.825
Defocus range (µm)	-2.0 to -0.6	-2.0 to -0.6	-2.0 to -0.6	-2.0 to -0.6	-2.0 to -0.6	-2.0 to -0.6
Defocus step (µm)	-0.2	-0.2	-0.2	-0.2	-0.2	-0.2
Symmetry imposed	None (C1)	None (C1)	None (C1)	None (C1)	None (C1)	None (C1)
Initial particle images (number)	3,961,114 + 1,343,714			3,342,145		
Final particle images (number)	70502			146537		
FSC threshold	0.143	0.143	0.143	0.143	0.143	0.143
Map resolution (Å)	3.06	2.79	2.66	2.93	2.61	2.59
**Refinement**						
CC (mask)	0.8	0.92	0.93	0.63	0.71	0.85
Resolution estimates (Å)						
d 99						
Masked	3.7	3.4	3.2	3.2	3.3	3.2
Unmasked	3.5	3.2	3.1	3.1	3.1	3.0
d FSC model, 0/0.143/0.5 (Å)						
Masked	3.0/3.0/3.4	2.7/2.8/3.0	2.6/2.6/2.8	2.9/2.9/3.6	2.5/2.6/3.1	2.5/2.6/2.8
Unmasked	3.0/3.0/3.8	2.8/2.8/3.3	2.6/2.7/3.2	2.9/3.1/4.1	2.5/2.7/3.4	2.6/2.6/3.0
Model composition						
Protein residues	4698	2358	2552	4717	2276	2449
Non-hydrogen atoms	38186	19437	20425	38164	18397	19678
Ligands	39	24	15	32	20	14
Water	428	166	258	416	184	296
**B factors (Å^2)**						
	Min/max/mean					
Protein residues	19.42/170.42/59.36	29.16/207.45/66.59	19.42/185.48/59.79	0.01/130.10/22.09	0.01/130.10/21.75	0.64/110.12/22.92
Ligands	30.00/175.57/59.36	37.72/175.57/88.66	30.00/73.33/48.65	1.32/93.30/43.00	8.29/79.35/44.14	1.32/93.30/35.91
water	29.39/104.02/55.75	40.80/104.02/67.30	29.39/93.14/48.44	0.07/78.52/17.44	0.07/65/55/20/09	0.91/78.52/14.43
**Validation**						
Ramachandran plot						
Favoured (%)	96.12	95.67	96.24	95.94	96.12	96.26
Allowed (%)	3.84	4.29	3.76	4.04	3.84	3.70
Disallowed (%)	0.04	0.04	0.00	0.02	0.04	0.04

Supplementary Table 5 | List of MD simulations.

Simulation	System	E-channel state	Started from	Time (ns)
**S1/S2**	WT	E157$^-$/**D79^0**/E36$^-$/E72$^-$	-	2×500
**S3/S4**	WT	**E157^0**/**D79^0**/E36$^-$/E72$^-$	-	2×500
**S5/S6**	D79N[A]	**E157^0**/ E36$^-$/E72$^-$	-	2×500
**S7/S8**	WT	E157$^-$/D79$^-$/**E36^0**/E72$^-$	S1/S2	2×100
**S9/S10**	WT	**E157^0**/D79$^-$/**E36^0**/E72$^-$	S3/S4	2×100
**S11/S12**	WT	E157$^-$/**D79^0**/**E36^0**/E72$^-$	S9/S10	2×100
**S13/S14**	WT	E157$^-$/D79$^-$/E36$^-$/**E72^0**	S7/S8	2x100
**S15/S16**	WT	**E157^0**/D79$^-$/E36$^-$/E72$^-$	-	2x100
**S17/S18**	D79N[A]	E157$^-$/ E36$^-$/E72$^-$	-	2x100
			**Total**	3.6 μs

Supplementary Table 6 | List of QM/MM simulations.

Simulation	System	E-channel state	QM region	Total sampling (ps)
**Q1**	WT	$E157^{0}/D79^{0\,-}$	$D79^{A}$, $E36^{K}$, $E157^{H}$, $F75^{A}$, $V76^{A}$, $T153^{H}$, $Y156^{H}$, $V58^{J}$, $Y59^{J}$, $A62^{J}$, $N40^{K}$, $A73^{K}$, 24 waters	65 × (8 ps) = 520 ps
**Q2**	WT	$E157^{-}/D79^{0}$	$D79^{A}$, $E36^{K}$, $E157^{H}$, $F75^{A}$, $V76^{A}$, $T153^{H}$, $Y156^{H}$, $V58^{J}$, $Y59^{J}$, $A62^{J}$, $N40^{K}$, $A73^{K}$, 23 waters	41 × (8 ps) = 328 ps
**Q3**	$D79N^{A}$	$E157^{0\,-}$	$N79^{A}$, $E36^{K}$, $E157^{H}$, $F75^{A}$, $V76^{A}$, $T153^{H}$, $Y156^{H}$, $V58^{J}$, $Y59^{J}$, $A62^{J}$, $N40^{K}$, $A73^{K}$, 26 waters	63 × (6 ps) = 378 ps
			**Total:**	**1226 ps**

Supplementary Table 7. Rate constants (in s^{-1}) calculated for different driving forces (with T=310 K), used in the kinetic models (see Supplementary Fig. 13).

			$\Delta G_{E157 \rightarrow D79}$ (kcal mol^{-1})					
			0.0	-1.0	-2.0	-3.0	-4.0	-5.0
$\Delta G_{D79 \rightarrow E36}$ (kcal mol^{-1})	k_{-1}	$k_2 = 10k_{-1}$	k_{-2}					
0.0	1.00	10.00	10.00	1.97	0.39	0.077	0.015	0.0030
1.0	5.07	50.66	50.66	10.00	1.97	0.39	0.077	0.015
2.0	25.66	256.62	256.62	50.66	10.00	1.97	0.39	0.077
3.0	130.00	1299.97	1299.97	256.62	50.66	10.00	1.97	0.39
4.0	658.53	6585.33	6585.33	1299.97	256.62	50.66	10.00	1.97
5.0	3335.97	33359.70	33359.70	6585.33	1299.97	256.62	50.66	10.00
			$\Delta G_{E157 \rightarrow D79}$ (kcal mol^{-1})					
			0.0	-1.0	-2.0	-3.0	-4.0	-5.0
$\Delta G_{D79 \rightarrow E36}$ (kcal mol^{-1})	k_{-1}	$k_2 = k_{-1}$	k_{-2}					
0.0	1.00	1.00	1.00	0.20	0.039	0.0077	0.0015	0.00030
1.0	5.07	5.07	5.07	1.00	0.20	0.04	0.0077	0.0015
2.0	25.66	25.66	25.66	5.07	1.00	0.20	0.039	0.0077
3.0	130.00	130.00	130.00	25.66	5.07	1.00	0.20	0.039
4.0	658.53	658.53	658.53	130.00	25.66	5.07	1.00	0.20
5.0	3335.97	3335.97	3335.97	658.53	130.00	25.66	5.07	1.00

Supplementary references

1. Sievers, F. *et al.* Fast, scalable generation of high-quality protein multiple sequence alignments using Clustal Omega. *Mol. Syst. Biol.* **7**, 539 (2011).
