# Supplementary material for: A carboxylate switch point controls long-range energy transduction in respiratory Complex I
Source: Nat Commun. 2026 Jul 1;17:5737. doi: 10.1038/s41467-026-74767-6 (PMC13324432; doi:10.1038/s41467-026-74767-6)
Supplement: Supplementary file 2 — Description of Additional Supplementary Files [file 41467_2026_74767_MOESM2_ESM.pdf]

Description of Additional Supplementary Files

File name: Supplementary Movie 1
Description: Proton transfer along the E-channel of Complex I.

File name: Supplementary Movie 2
Description: Proton transfer along the E-channel of the $D79N^A$ variant.
