# Supplementary material for: A carboxylate switch point controls long-range energy transduction in respiratory Complex I
Source: Nat Commun. 2026 Jul 1;17:5737. doi: 10.1038/s41467-026-74767-6 (PMC13324432; doi:10.1038/s41467-026-74767-6)
Supplement: Supplementary file 5 — Reporting summary [file 41467_2026_74767_MOESM5_ESM.pdf]

nature portfolio

Corresponding author(s): Ville R. I. Kaila

Last updated by author(s): Sep 15, 2025

# Reporting Summary

Nature Portfolio wishes to improve the reproducibility of the work that we publish. This form provides structure for consistency and transparency in reporting. For further information on Nature Portfolio policies, see our Editorial Policies and the Editorial Policy Checklist.

## Statistics

For all statistical analyses, confirm that the following items are present in the figure legend, table legend, main text, or Methods section.

| n/a | Confirmed | |
| --- | --- | --- |
| ☐ | ☒ | The exact sample size (*n*) for each experimental group/condition, given as a discrete number and unit of measurement |
| ☐ | ☒ | A statement on whether measurements were taken from distinct samples or whether the same sample was measured repeatedly |
| ☒ | ☐ | The statistical test(s) used AND whether they are one- or two-sided<br>*Only common tests should be described solely by name; describe more complex techniques in the Methods section.* |
| ☒ | ☐ | A description of all covariates tested |
| ☒ | ☐ | A description of any assumptions or corrections, such as tests of normality and adjustment for multiple comparisons |
| ☐ | ☒ | A full description of the statistical parameters including central tendency (e.g. means) or other basic estimates (e.g. regression coefficient) AND variation (e.g. standard deviation) or associated estimates of uncertainty (e.g. confidence intervals) |
| ☒ | ☐ | For null hypothesis testing, the test statistic (e.g. *F*, *t*, *r*) with confidence intervals, effect sizes, degrees of freedom and *P* value noted<br>*Give P values as exact values whenever suitable.* |
| ☒ | ☐ | For Bayesian analysis, information on the choice of priors and Markov chain Monte Carlo settings |
| ☒ | ☐ | For hierarchical and complex designs, identification of the appropriate level for tests and full reporting of outcomes |
| ☒ | ☐ | Estimates of effect sizes (e.g. Cohen's *d*, Pearson's *r*), indicating how they were calculated |

*Our web collection on statistics for biologists contains articles on many of the points above.*

## Software and code

Policy information about availability of computer code

| | |
| --- | --- |
| Data collection | cryo-EM: Smart EPU v2.12.1, Fluorescence spectra: Cary Eclipse Fluorescence Spectrophotometer (Agilent Technologies), UV-vis spectra: Ocean-FX-UV-VIS-ES (Ocean Insight), SEC: ÄKTA Pure 25M1 fast performance liquid chromatography, MD: NAMD v2.14/3.06b, QM/MM: FermiONs++ |
| Data analysis | Cryosparc v4.4.0, CTFFIND4, Phenix 1.20.1, Coot 0.9.8.3, MolProbity, VMD 1.93-1.94, UCSF ChimeraX 1.10, NAMD2.14, NAMD3.06b, FermiONs++, MDAnalysis, COPASI 4.35, TUPA, Python/Matplotlib/NumPy/SciPy, CAVER |

For manuscripts utilizing custom algorithms or software that are central to the research but not yet described in published literature, software must be made available to editors and reviewers. We strongly encourage code deposition in a community repository (e.g. GitHub). See the Nature Portfolio guidelines for submitting code & software for further information.

## Data

Policy information about availability of data

All manuscripts must include a data availability statement. This statement should provide the following information, where applicable:

- Accession codes, unique identifiers, or web links for publicly available datasets
- A description of any restrictions on data availability
- For clinical datasets or third party data, please ensure that the statement adheres to our policy

CryoEM maps are available in the Electron Microscopy Data Bank (EMDB ID: EMD-55748, EMD-55749, EMD-55750, EMD-55751, EMD-55752, and EMD-55753), and

atomic models are available in the Protein Data Bank (PDB ID: 9TAJ, 9TAK, 9TAL, 9TAM, 9TAN and 9TAO). Models and simulation setups have been deposited to the Zenodo database under: 10.5281/zenodo.17122031. Data provided is provided in the source data file.

## Research involving human participants, their data, or biological material

Policy information about studies with human participants or human data. See also policy information about sex, gender (identity/presentation), and sexual orientation and race, ethnicity and racism.

| | |
|---|---|
| Reporting on sex and gender | N/A |
| Reporting on race, ethnicity, or other socially relevant groupings | N/A |
| Population characteristics | N/A |
| Recruitment | N/A |
| Ethics oversight | N/A |

Note that full information on the approval of the study protocol must also be provided in the manuscript.

# Field-specific reporting

Please select the one below that is the best fit for your research. If you are not sure, read the appropriate sections before making your selection.

☒ Life sciences ☐ Behavioural & social sciences ☐ Ecological, evolutionary & environmental sciences

For a reference copy of the document with all sections, see nature.com/documents/nr-reporting-summary-flat.pdf

# Life sciences study design

All studies must disclose on these points even when the disclosure is negative.

| | |
|---|---|
| Sample size | All experiments were conducted at least in biological duplicates which are tested in technical triplicates. The spectrosopic data were collected from n=6-9 independent measurements.<br>WT Complex I cryo-EM: 3,961,114 Mio (dataset 1) and 1,343,714 Mio particles (dataset 2) were collected for 2D classification, reduced to 63,982 / 46,649 particles with further 2D classification of the merged to 90 534 particles, and assigned into two classes. Further non-uniform and local refinement resulted in the final map.<br>D79N Complex I cryoEM: 3,342,145 Mio particles were collected for 2D classification, reduced to 530,915 particles with further 2D classification, followed by selection of 192869 particles, and assigned into two classes. Further non-uniform and local refinement resulted in the final map.<br>MD simulations were run in duplicates, with data collected over 100-500 ns trajectories. QM/MM free energy calculations were performed over 328-520 ps simulations, with statistical errors analysed using bootstrapping analysis. |
| Data exclusions | No data were excluded from the biochemical, structural or computational experiments. 2D image templates were generated by manual picking. Particles belonging to classes with "broken" protein were removed from the analysis. |
| Replication | Proton pumping measurements were performed in n=6 independent replicates. Each cryoEM map for WT Complex I was generated based on two data set. MD simulations were run in two replicas. Analysis of MD properties were carried out using one snapshot per ns. QM/MM simulations were carried out on a single, selected snapshot from MD simulations. |
| Randomization | Not applicable. |
| Blinding | Not applicable. |

# Reporting for specific materials, systems and methods

We require information from authors about some types of materials, experimental systems and methods used in many studies. Here, indicate whether each material, system or method listed is relevant to your study. If you are not sure if a list item applies to your research, read the appropriate section before selecting a response.

## Materials & experimental systems

| n/a | Involved in the study |
|---|---|
| ☒ | ☐ Antibodies |
| ☒ | ☐ Eukaryotic cell lines |
| ☒ | ☐ Palaeontology and archaeology |
| ☒ | ☐ Animals and other organisms |
| ☒ | ☐ Clinical data |
| ☒ | ☐ Dual use research of concern |
| ☒ | ☐ Plants |

## Methods

| n/a | Involved in the study |
|---|---|
| ☒ | ☐ ChIP-seq |
| ☒ | ☐ Flow cytometry |
| ☒ | ☐ MRI-based neuroimaging |

# Plants

| | |
|---|---|
| Seed stocks | N/A |
| Novel plant genotypes | N/A |
| Authentication | N/A |
