# Supplementary material for: A carboxylate switch point controls long-range energy transduction in respiratory Complex I
Source: Nat Commun. 2026 Jul 1;17:5737. doi: 10.1038/s41467-026-74767-6 (PMC13324432; doi:10.1038/s41467-026-74767-6)
Supplement: Supplementary file 6 — Transparent Peer Review file [file 41467_2026_74767_MOESM6_ESM.pdf]

nature portfolio

Peer Review File

A Carboxylate Switch Point Controls Long-Range Energy Transduction in Respiratory Complex I

Corresponding Author: Professor Ville Kaila

This manuscript has been previously reviewed at another journal. This document only contains information relating to versions considered at Nature Communications.

Version 0:

Reviewer comments:

Reviewer #1

(Remarks to the Author)
The authors have satisfactorily addressed all of my previous comments.

Reviewer #2

(Remarks to the Author)
I read over the revision and response to the questions that I raised in the previous round of review. I believe the authors have largely addressed my questions. Even with the reduction of PCET activity to 20%, the impact is orders of magnitude smaller by comparison to the decrease of the PT rate constant that the computations have predicted. Nevertheless, in the grand scheme of things, this is a relatively minor point. I believe the work makes a valuable contribution to the bioenergetics field.

Reviewer #3

(Remarks to the Author)
The authors have addressed my previous concerns adequately, therefore I suggest acceptance of the manuscript.

Open Access This Peer Review File is licensed under a Creative Commons Attribution 4.0 International License, which permits use, sharing, adaptation, distribution and reproduction in any medium or format, as long as you give appropriate credit to the original author(s) and the source, provide a link to the Creative Commons license, and indicate if changes were made.
In cases where reviewers are anonymous, credit should be given to 'Anonymous Referee' and the source.
The images or other third party material in this Peer Review File are included in the article's Creative Commons license, unless indicated otherwise in a credit line to the material. If material is not included in the article's Creative Commons license and your intended use is not permitted by statutory regulation or exceeds the permitted use, you will need to obtain permission directly from the copyright holder.
To view a copy of this license, visit https://creativecommons.org/licenses/by/4.0/
